# Prise en charge diagnostique et thérapeutique de la tuberculose ganglionnaire en Tunisie

**DOI:** 10.11604/pamj.2014.19.211.5213

**Published:** 2014-10-27

**Authors:** Hajer Ben Brahim, Ikbel Kooli, Abir Aouam, Adnene Toumi, Chawki Loussaief, Jamel Koubaa, Mohamed Chakroun

**Affiliations:** 1 Service de Maladies Infectieuses, CHU Fattouma Bourguiba, 5000 Monastir, Tunisie; 2 Service d'Oto-Rhino-Laryngologie, CHU Fattouma Bourguiba, 5000 Monastir, Tunisie

**Keywords:** Tuberculose ganglionnaire, cytoponction, biopsie, chirurgie, traitement, lymph node tuberculosis, fine needle aspiration, biopsy, surgery, treatment

## Abstract

La tuberculose ganglionnaire est la localisation extra-pulmonaire la plus fréquente de la tuberculose. Nous nous proposons dans ce travail d’étudier les modalités diagnostiques, thérapeutiques et évolutives de cette localisation. Il s'agit d'une étude rétrospective portant sur 100 cas de tuberculose ganglionnaire. L’âge moyen était de 35 ± 15 ans (15-85 ans). Aucun malade n’était VIH positif. L'aire cervicale était la plus touchée (93 cas). L'intradermo-réaction à la tuberculine était positive dans 76/91 cas (83,5%). L'examen bactériologique des prélèvements au niveau des ganglions atteints avait mis en évidence des bacilles acido-alcoolo-résistants à l'examen direct dans 2/31 cas (6,4%) et la culture avait isolé *Mycobacteruim tuberculosis* dans 1/31 cas (3,2%). La cytoponction ganglionnaire (FNAC) était évocatrice de tuberculose dans 35/42 cas (83,3%). La biopsie ganglionnaire était réalisée dans 69 cas et avait permis de retenir le diagnostic de tuberculose dans tous les cas. La FNAC, comparativement à la biopsie, avait permis de raccourcir significativement le délai de la prise en charge (15,1 vs 22,8 jours; p=0,001) et la durée d'hospitalisation (17,3 vs 24,6; p=0,004). La durée moyenne du traitement antituberculeux était de 9,8 ± 4,6 mois (7 à 44 mois). Le traitement chirurgical initial avait raccourci significativement la durée du traitement médical. Il n'avait pas d'impact sur le taux de guérison. Nous avons noté 10 cas de réponse paradoxale aux antituberculeux, quatre cas de résistance clinique et une rechute dans deux cas. La tuberculose ganglionnaire pose un problème diagnostique et thérapeutique. La microbiologie est d'un faible apport. La FNAC est un moyen diagnostique très utiles dans les pays endémiques et à faibles ressources. Un traitement médical seul permet d’éviter les inconvénients de la chirurgie.

## Introduction

L'atteinte ganglionnaire représente la localisation extra pulmonaire la plus fréquente de la tuberculose [1, 2]. Elle pose jusqu’à nos jours des difficultés en rapport avec la stratégie diagnostique et thérapeutique à suivre et les modalités évolutives surtout dans les pays endémiques tel que la Tunisie.

## Méthodes

Nous avons étudié rétrospectivement les dossiers des malades ayant été hospitalisés pour une tuberculose ganglionnaire dans les services de Maladies Infectieuses et d'ORL du CHU Fattouma Bourguiba de Monastir au cours de la période allant du 1er janvier 1992 au 31 décembre 2010 Tous les malades qui avaient une tuberculose ganglionnaire confirmée et qui étaient régulièrement suivis pendant la période de l’étude étaient inclus dans notre travail. Une durée minimale de 7 mois de suivi sous traitement antituberculeux était exigée. Tous les malades avaient bénéficié d'un examen histologique du ganglion atteint, soit par cytoponction ganglionnaire (FNAC), quand les moyens techniques sont disponibles, soit par biopsie. Dans les cas ou la cytoponction ganglionnaire était non concluante, une biopsie était réalisée.

Le diagnostic de tuberculose ganglionnaire est considéré certain, si le malade répond aux critères diagnostiques sous cités, sinon il est considéré probable: mise en évidence de bacille acido-alcoolo-résistant (BAAR) par la technique classique de Ziehl-Neelsen dans le liquide de ponction ou tissu ganglionnaire après biopsie ou pus provenant d'une fistule; et/ou: découverte à l'examen cytologique ou anatomopathologique d'un prélèvement ganglionnaire d'une nécrose caséeuse et/ou de granulomes épithéloïdes et gigantocellulaires; et dans les deux cas, une réponse favorable au traitement antituberculeux.

N’étaient pas inclus dans l’étude les malades âgés de moins de 15 ans et/ou perdus de vue précocement et chez qui le suivi thérapeutique était inférieur à la durée minimale de 7 mois. Le but de ce travail était d’étudier les modalités diagnostiques, thérapeutiques et évolutives de la tuberculose ganglionnaire.

## Résultats

Cent malades, répondant aux critères d'inclusion, étaient retenus dans l’étude. La tuberculose ganglionnaire était certaine dans 96 cas et probable dans 4 cas. L’âge moyen des malades était de 35 ± 15 ans (15-85 ans) avec un sexe ratio de 0,5. Un antécédent personnel de tuberculose était noté dans 3 cas et un contage tuberculeux dans 12 cas. Aucun malade n’était infecté par le VIH. L’ aire cervicale était la plus touchée (93 cas). Une atteinte des ganglions profonds était notée dans 6 cas. L'association concomitante à un autre foyer tuberculeux était notée dans 8 cas dont l'atteinte pulmonaire était la plus fréquente (3 cas).

Sur le plan microbiologique, 242 prélèvements étaient examinés, dont 31 au niveau du ganglion atteint. L'examen direct avait mis en évidence la présence de BAAR dans 3/242 prélèvements (1,2%) et la culture avait permis d'isoler le BK dans 1/242 prélèvements (0,4%) (Tableau 1). La FNAC était pratiquée dans 42 cas. Elle avait permis de retenir le diagnostic dans 35 cas (83,3%), en montrant la présence de nécrose caséeuse et/ou de granulomes, et était non concluante dans 4 cas (9,5%) (Tableau 2). La biopsie ganglionnaire était pratiquée dans 69 cas, dont 11 cas avaient bénéficié d'une FNAC de première intention. Elle avait permis de poser le diagnostic dans tous les cas. Les principales anomalies histologiques sont résumées dans le Tableau 3. La rentabilité de la biopsie ganglionnaire dans le diagnostic histologique de la tuberculose était meilleure que la cytoponction (100% vs 83,3%). La comparaison des deux moyen de diagnostic histologique avait montré que la FNAC avait raccourci de façon significative le délai de prise en charge thérapeutique (15,1 vs 22,8 jours; p=0,001) et la durée d'hospitalisation (17,3 vs 24,6 jours; p=0,004).

**Tableau 1:** Résultats de l’étude bactériologique

Produits de recherche	Nombre de prélèvements	Examen direct		Culture	
		+	-	+	-
Crachats	165	1	164	0	165
Liquides de ponction ganglionnaire	7	1	6	1	6
Broyats ganglionnaires	24	1	23	0	24
Urines	45	0	45	0	45
LCR	1	0	1	0	1
TOTAL	242	3	239	1	241

**Tableau 2:** Fréquence des différentes anomalies cytologiques à la FNAC

Anomalie	Nombre de cas	Fréquence (%)
Granulomes avec nécrose caséeuse	26	61,9
Nécrose caséeuse sans granulomes	5	11,9
Granulomes sans nécrose caséeuse	4	9,5
Infiltrat inflammatoire	2	4,8
Lymphadénite aiguë suppurée	1	2,4
Non concluante	4	9,5
TOTAL	42	100

**Tableau 3:** Fréquence des différentes anomalies histologiques à la FNAC et à la biopsie ganglionnaire

Anomalie	Cytoponction	Biopsie	Total
Granulomes avec nécrose caséeuse	26	61	87
Nécrose caséeuse sans granulomes	5	3	8
Granulomes sans nécrose caséeuse	4	5	9
Infiltrat inflammatoire	3	_	3
Non concluantes	4	_	4
Total	42	69	111

Sur le plan thérapeutique, le délai moyen de prise en charge était de 19,5 ± 12 jours (9 - 90 j). Les malades recevaient une première phase de traitement dite de quadrithérapie d'une durée moyenne de 2,5 ± 0,9 mois (2 - 4 mois). Au cours de cette phase, l'association Isoniazide, Rifampicine, Pyrazinamide et Ethambutol était prescrite dans 81 cas. La deuxième phase de continuation, dite de bithérapie, était de durée moyenne de 7,2 ± 2 mois (5 - 17 mois). Au cours de cette phase l'association Rifampicine et Isoniazide était prescrite dans 95 cas. Les différentes molécules utilisées sont représentées dans le Tableau 4. La durée totale du traitement antituberculeux était de 9,8 ± 4,6 mois (7-44 mois).

**Tableau 4:** Fréquence des différents antituberculeux utilisés

Molécule	Nombre de malades
Isoniazide	100
Rifampicine	95
Pyrazinamide	96
Ethambutol	89
Streptomycine	15
Ciprofloxacine	2
Ethionamide	1
Amikacine	1

Le recours à la chirurgie était indiqué dans 55 cas. Parmi ces malades, 38 (69%) avaient subi une biopsie exérèse totale de l'adénopathie, dans un but diagnostique avant de commencer le traitement médical. Dans les 17 autres cas (31%), la chirurgie était à but thérapeutique, suite aux complications des adénopathies. La comparaison des malades ayant bénéficié d'une biopsie exérèse initiale (38 cas) et ceux traités médicalement avait montré un taux de guérison de 100% dans les deux groupes et une durée du traitement médical significativement plus longue dans le deuxième groupe (8,7 vs 10,6 mois; p=0,017).

Le suivi clinique et thérapeutique était complet pour 59 malades avec un recul moyen de 19 ± 18,1 mois (1 - 84 mois) après la fin du traitement. Chez ces malades, une guérison était obtenue dans tous les cas. Trente neuf malades étaient perdus de vue avant la fin du traitement. Pour ces malades la guérison apparente était notée, au dernier contrôle, dans 37 cas (94,8%). Une rechute de la maladie était notée dans deux cas. Quatre malades avaient présenté une résistance clinique aux antituberculeux dits de première ligne sans pouvoir la documenter bactériologiquement sur les différents prélèvements. Chez ces malades une mauvaise observance au traitement était retrouvée dans deux cas. Une réponse paradoxale au traitement antituberculeux était notée dans 10 cas après un délai moyen de 4,5 mois (10 jours-12 mois).

## Discussion

La tuberculose demeure un fléau infectieux majeur, particulièrement dans les pays en voie de développement [3, 4]. Dans 25% des cas, la localisation de la tuberculose est extrapulmonaire dont l'atteinte ganglionnaire est la plus fréquente [2, 5, 6]. L'adénopathie tuberculeuse est le plus souvent unilatérale, unique et isolée [7]. La localisation cervicale est de loin la plus fréquente [8, 9]. Dans la littérature, on retrouve une association à d'autre localisations tuberculeuses rapportée dans 36 à 58% des cas [7, 10]. Parmi ces localisations, l'atteinte pulmonaire reste la plus fréquente. Dans notre série, le faible pourcentage de tuberculose pulmonaire associée pourrait être expliqué par le fait que le bilan tuberculeux n’était pas pratiqué de façon systématique pour tous nos malades. La confirmation diagnostique repose sur l'isolement de Mycobacteruim tuberculosis dans le ganglion atteint. Cependant, le taux de positivité de la recherche de BK à l'examen direct, des produits de ponction ganglionnaire, ne dépasse pas 37,4% des cas dans les différentes séries. Celui de la culture est plus élevé variant entre 19 et 71% des cas [11, 12].

Notre étude signale le très faible apport de la bactériologie et surtout de la culture du BK dans notre hôpital par rapport aux différentes séries. Ceci peut être expliqué part un défaut technique au niveau du laboratoire du à des conditions inadéquates de recueil, d'acheminement et de conservation des prélèvements. La FNAC joue un rôle important dans le diagnostic étiologique des adénopathies [7, 13]. C'est une procédure simple, rapide, fiable, non invasive et peu coûteuse [7, 14]. La rentabilité diagnostique de la FNAC, dans les différentes séries, varie de 50 à 92,8%. Ce rendement, opérateur dépendant, peut être amélioré en associant les résultats de la cytoponction à ceux de l'IDR [8]. Dans notre série, elle était de 83,3%. De l'autre part, la biopsie ganglionnaire constitue le « Gold Standard » du diagnostic histologique de la tuberculose ganglionnaire [15–17]. Elle reste une méthode plus sensible que la FNAC puisque elle explore la quasitotalité du ganglion. Cette attitude nécessite au moins la réalisation d'une adénectomie qui constitue tout de même un acte chirurgical, réalisé souvent sous anesthésie générale, et comportant un risque. Le coût de l'acte, le retard diagnostique possible lié aux rendez-vous opératoires lointains, le taux élevé de fistulisation et de rechute et le côté inesthétique de la cicatrice opératoire viennent se surajouter aux inconvénients de cette méthode diagnostique [18, 19] Dans notre étude, la cytoponction avait une rentabilité inférieure à celle de la biopsie (83,3 Vs 100%) mais elle avait permis de raccourcir significativement le délai de prise en charge thérapeutique et d'hospitalisation. Ces résultats rendent la FNAC un examen très utiles pour le diagnostic de la tuberculose ganglionnaire particulièrement dans les pays en voie de développement, à faibles ressources et à forte endémicité tuberculeuse [14, 18, 20].

La revue de la littérature ne trouve pas de consensus ni d’étude de qualité permettant de conclure sans controverse sur une durée optimale du traitement de la tuberculose ganglionnaire. Selon l'OMS, le Conseil supérieur d'hygiène publique en France (CSHPF) et la Société de pneumologie de langue française (SPLF), une durée totale de six mois est jugée suffisante [21, 22]. A travers les différentes séries publiées on constate que la majorité des praticiens, traitaient les tuberculoses ganglionnaires pendant des durées nettement supérieures à 6 mois [23, 24]. Des études supplémentaires sont nécessaires pour établir un consensus et standardiser la durée de traitement de la tuberculose ganglionnaire comme c'est le cas pour la tuberculose pulmonaire.

## Conclusion

La chirurgie au cours de la tuberculose ganglionnaire n'est pas dénuée de complications, surtout dans les localisations cervicales. Dans notre étude le taux de guérison était le même dans les deux groupes avec et sans chirurgie. La biopsie exérèse chirurgicale est nécessaire si les moyens diagnostiques non invasifs, particulièrement la cytologie, n’étaient pas contributifs et lorsqu'une affection néoplasique est suspectée. Dans les autres cas, la chirurgie doit être évitée et indiquée que devant des formes compliquées.
